# Process design of industrial-scale membrane distillation system for wastewater treatment in nano-electronics fabrication facilities

**DOI:** 10.1016/j.mex.2020.101066

**Published:** 2020-09-17

**Authors:** Imtisal-e- Noor, Andrew Martin, Olli Dahl

**Affiliations:** a Department of Energy Technology, KTH Royal Institute of Technology, Stockholm, Sweden; b Department of Bioproducts and Biosystems, Aalto University, Espoo, Finland

**Keywords:** Equipment design, Industrial-scale, Economic analysis, Mass and energy balances, Membrane distillation, AGMD, Air Gap Membrane Distillation, CAPEX, Capital Expenditures, CEPCI, Chemical Engineering Plant Cost Index, CF, Contingency Fees, CO, Construction Overhead, I, Insurance, ISBL, Inside Battery Limits, MD, Membrane Distillation, NC, Normalized Capital Investment, NO, Normalized Operating and Maintenance Investment, OPMEX, Operating and Maintenance Expenditure, OSBL, Outside Battery Limits, STEC, Specific Thermal Energy Consumption, TCI, Total Capital Investment, TDC, Total Depreciable Capital, TPC, Total Permanent Capital, VOC, Volatile Organic Compound, WC, Working Capital

## Abstract

The main challenge for implementing an industrial-scale membrane distillation (MD) system is its associated thermal power demand and resulting operational cost, which hinders the commercialization of the technology, even after forty years of its evolution and development. Nevertheless, an enormous amount of waste heat releasing from the nano-electronics facilities provides MD an opportunity to showcase its potential for treating industrial wastewater discharging from the facilities. In this work, a waste heat driven MD system for a plant capacity of 15 m^3^/h was analyzed in terms of its thermal power demand and unit wastewater treatment cost. The economic analysis was performed using the factored estimate method. The results show that the thermal power requirement of the industrial-scale MD system was 12.38 MW, and the unit water treatment cost can vary between 3-23 $/m^3^, based on plant type (i.e., retrofitted facility or new wastewater treatment facility).

• Determination of various industrial waste heat sources in typical nano-electronics fabrication facilities via interviews of related professionals, and designed industrial-scale waste heat integrated MD system for nano-electronics industries
• Mass and energy balances around the industrial-scale MD system for wastewater treatment in nano-electronics industries
• Equipment design for the purpose and performed economic evaluation of the MD system by customizing factored estimate method

## Graphical abstract

**Figure:**
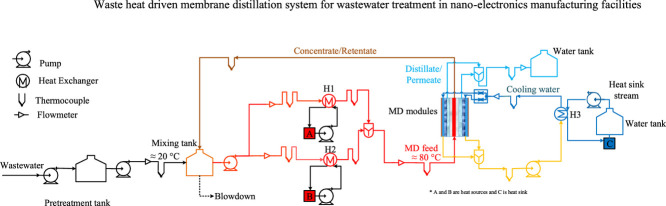

Specifications Table

**Table:** 

Subject Area:	Chemical Engineering
More specific subject area:	Membrane Engineering
Method name:	System analysis of Membrane Distillation
Name and reference of the original method:	D. W. Green and R. H. Perry, Perry's Chemical Engineers’ Handbook, Eighth Edition. McGraw-Hill Education, 2007.

## 1. Background

**Fig. 1:**
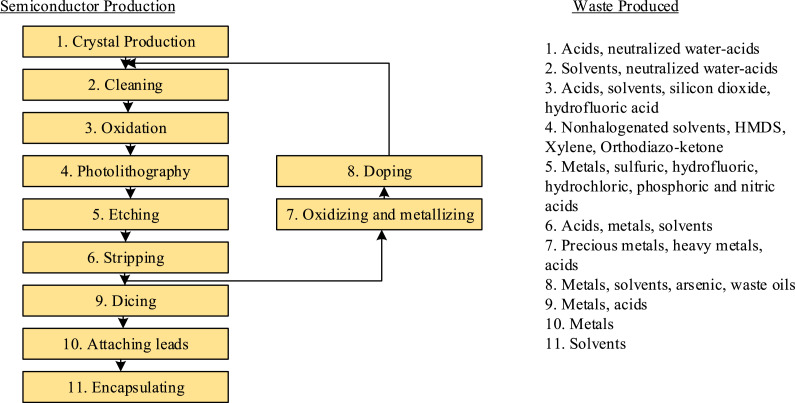
Waste produced in nano-electronics fabrication facilities (adapted from [2]).

Nano-electronics industries have very complex and delicate processes for the fabrication of semiconductor chips, that require a large amount of ultrapure and process water. Typically, nano-electronics industries consume ~1000 m^3^/day of ultrapure water [1]. Consequently, the manufacturing units generate a corresponding amount of wastewater. These waste water streams include acids, spent solvents, waste oils, and metals [2]. Fig. 1 represents the waste produced in a typical nano-electronics manufacturing plant. Direct discharge of these wastewaters into a water body is strictly regulated by water quality control authorities unless they are pretreated according to international and local standards. Current emphasis is being placed on reducing the amount of waste produced. However, until a more suitable means of production is established, the waste streams must be treated.

**Fig. 2:**
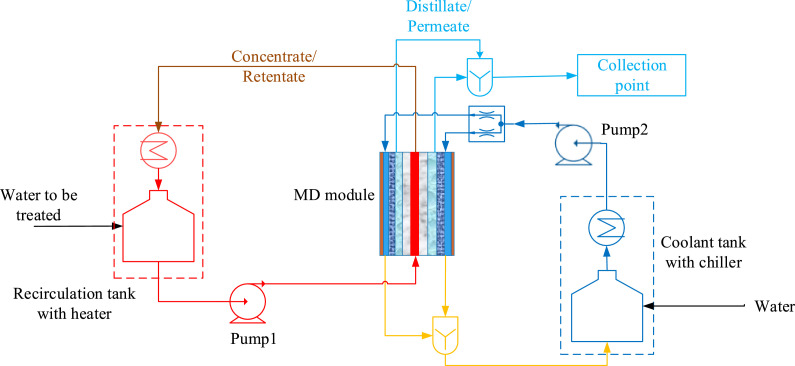
Schematic flow diagram of AGMD separation process.

In the present situation, there are many different technologies in use to treat wastewater involving mechanical separation to thermal separation. However, researchers are becoming attracted to employing membrane technologies due to their intrinsic environment-friendly attitude. Reverse osmosis, micro-filtration and nano-filtration are traditional membrane processes that are used for this purpose [3]. These methods are highly recognized and generally effective, but there is still a need for a cost-efficient system with enhanced water purity. Moreover, there are some technical issues involved in these technologies, especially organic and inorganic fouling, disposal of the brine solutions and upper pressurization limits. In this regard, “Membrane distillation” (MD) is one of the promising membrane processes which have unique characteristics for water treatment. The MD process takes place at temperatures below 100°C and at ambient pressure. As compared to other water purification methods, it is less sensitive to feed concentration, and it utilizes low-grade heat [4]. Fig. 2 presents the schematic diagram of the air gap membrane distillation (AGMD) separation process.

Due to the fact that the system can be operated using low-grade heat sources, there are many opportunities available as heat sources, including district heating [5] and industrial waste heat [6]. These integrated systems provide sustainable, cost-effective and energy-efficient approaches for wastewater treatment. Moreover, these systems can play a vital role in reducing carbon footprints and promoting the concept of a green world. In this work, industrial waste heat integrated membrane distillation process for wastewater treatment in nano-electronics fabrication facilities is investigated.

### 2. Method details

Previously published works [7,8] clearly indicate the successful application of MD in nano-electronics fabrication facilities for the treatment of different wastewater streams. However, the detailed mass and energy balances and economic evaluations are not provided for verification of industrial-scale MD system. Woldemariam et al. [5] presented a study on a detailed parametric analysis of a semi-commercial Xzero MD module, including temperature and flow rate dependencies on permeate yield and thermal energy demand; therefore, it is considered as a reference for performing the technical evaluations. This work is performed for a wastewater treatment plant having a capacity of 15 m^3^/h. (This paper is a co-submission of original research presented in [9]). Different industrial waste heat sources can be considered for operating an industrial-scale MD system in typical nano-electronics facilities. These heat sources are as follows.

1. Condenser outlet water from chiller (35-90°C).
2. Process cooling water from manufacturing tools (30-90°C).
3. Hot air from VOCs combustion abatement systems (350°C).
4. Spent etchant (phosphoric acid) from nitride etching (150-200°C).
5. Spent stripper (sulfuric acid) from photoresist stripping and cleaning (90-140°C).
6. Dissipated heat from compressors, steam generators and pumps (temperature varies significantly).

**Fig. 3:**
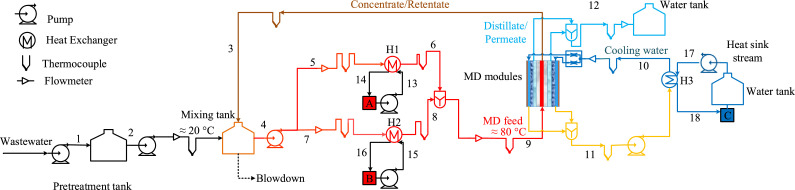
Flow diagram of proposed industrial-scale waste heat driven MD integrated system; A-Condenser outlet water from chiller; B- Hot air from VOCs abatement system; C- Heat sink.

These heat sources can collectively provide 35-40 MW of thermal power. It is noteworthy that the above-mentioned information is tightly held and not publicly available by official means. In this work, condenser outlet water from the chiller (8-12 MW) and hot air from VOCs combustion abatement system (0.25-0.5 MW) are chosen to provide the required thermal energy to the industrial-scale MD system. Fig. 3 shows the proposed waste heat driven MD system.

### 2.1. Mass and energy balance

Global mass and energy balances were performed in order to design process equipment for the integrated MD system. All calculations are performed considering atmospheric pressure.

*Commonly used symbols*

m = mass flow rate
T = temperature
Q  = thermal power
*c_p_* = heat capacity of the water
*c_pa_* = heat capacity of the air
A = area

The total thermal power demand (*Q_t_*) was calculated using Eq. (1).

(1) $$Q_{t}=\;m_{dT}STEC$$

where subscript *dT* depicts the desired total permeate, and specific thermal energy consumption is shown as STEC, which is taken from the reference study [5] when the operating conditions were as follows: MD feed inlet temperature = 80°C, cooling water inlet temperature = 26°C, and feed and coolant flow rates were 1200 L/h (0.333 kg/s).

$$Q_{t}=15\;m^{3}/h\times 825\;\text{kWh}/m^{3}=12.38\;\text{MW}.$$

Since the maximum power provided by heat exchanger H1 cannot more than 12 MW due to heat source thermal power limitation, therefore:

$$Q_{H1}=12\;\text{MW}$$

The remaining required power can be obtained considering heat exchanger H2, *Q*_*H*2_ can be calculated using Eq. (2).

(2) $$\begin{array}{c} Q_{H2}=Q_{t}-Q_{H1}\\ Q_{H2}=12.38\;\text{MW}-12\;\text{MW}\;=\;0.38\;\text{MW} \end{array}$$

Heat sources inlet streams temperatures (based on the information given above) are mentioned as follows:

$$\begin{array}{cc} T_{13} & =\;85{}^{\circ }C\\ T_{15}\; & =\;350{}^{\circ }C \end{array}$$

Feed, coolant and permeate temperature information was taken from the reference study and shown below.

$$\begin{array}{cc} T_{6} & =T_{8}=T_{9}=\;80{}^{\circ }C\\ T_{10} & =26{}^{\circ }C\\ T_{11} & =39{}^{\circ }C\\ T_{12} & =43{}^{\circ }C \end{array}$$

The equation used for calculating required MD feed flow rates based on the data obtained from the reference study is shown in Eq. (3).

(3) $$\frac{m_{dT}}{m_{d}}=\frac{m_{fT}}{m_{f}}$$

where subscript *fT* shows the total required MD feed and subscripts *d* and *f* are the reference permeate and MD feed when MD feed temperature was 80°C and cooling water temperature was 26°C.

Number of membrane modules in each reference cascade = 2
Reference membrane area for one MD cascade = A_m_ =4.6 m^2^
Reference MD feed flow rate for one MD cascade = 1200 L/h = 0.333 kg/s
Reference permeate flow rate for one MD cascade = 5.85 L/m^2^.h × 4.6 m^2^ × 1h/3600s = 0.0075 kg/s
Recovery ratio per cycle= 0.0075 kg/s / 0.33 kg/s = 0.0225
Desired permeate flow rate = 4.17 kg/s = 15 m^3^/h = 330 m^3^/day
Total MD feed flow rate = 4.17 kg/s /0.0225 = 185 kg/s = 666 m^3^/h = 14652 m^3^/day
In this work, it is assumed that 1kg of aqeous solution is equal to 1L of aqeous solution.
Thus,
  $$\begin{array}{c} m_{4}=m_{9}=185\;\text{kg}/s\\ m_{1}=m_{2}=m_{12}=4.17\;\text{kg}/s \end{array}$$

The equation used for global mass balance around MD is mentioned in Eq. (4).

(4) $$m_{fT}=\;m_{rT}+\;m_{dT}$$

where subscript *rT* is for the corresponding retentate.

$$m_{3}=\;185\;\text{kg}/s\;-\;4.17\;\text{kg}/s\;=\;180.83\;\text{kg}/s$$

The retentate temperature was 65°C for the reference conditions. Based on this information, the temperature after the mixing tank was calculated as using Eq. (5)

(5) $$\begin{array}{cc} T_{4} & =\frac{m_{2}c_{p}T_{2}+m_{3}c_{p}T_{3}}{m_{2}c_{p}+m_{3}c_{p}}\\ T_{1} & =\;T_{2}\;=\;20^{\circ }C\\ T_{3} & =65^{\circ }C\\ T_{4} & =\left(4.17\;\text{kg}/s\;\times 4180\;J/\text{kg}.^{\circ }C\;\times \;20^{\circ }C+\;180.83\;\text{kg}/s\;\times 4180\;J/\text{kg}.^{\circ }C\times 65^{\circ }C\right)/\\ & \;\left(4.17\;\text{kg}/s\times 4180\;J/\text{kg}.^{\circ }C+180.83\;\text{kg}/s\;\times 4180\;J/\text{kg}.^{\circ }C\right)\;T_{4}=T_{5}=T_{7}=63.98^{\circ }C \end{array}$$

The flow rate of the wastewater stream passing through the heat exchanger H1 can be calculated using Eq. (6).

(6) $$\begin{array}{c} m_{5}=m_{6}=\;\frac{Q_{H1}}{c_{p}\left(T_{6}-T_{5}\right)}\\ m_{5}=m_{6}=12\;\;\text{MW}/\left(4180\;J/\text{kg}.{}^{\circ }C\;\times \;\left(80{}^{\circ }C-63.98{}^{\circ }C\right)\right)=179.2\;\text{kg}/s \end{array}$$

The flow rate of the wastewater stream passing through the heat exchanger H2 can be calculated using Eq. (7).

(7) $$\begin{array}{c} m_{7}=m_{8}=m_{4}-m_{5}\\ m_{7}=m_{8}=185\;\text{kg}/s-179.32\;\text{kg}/s\;=\;5.67\;\text{kg}/s \end{array}$$

The pinch point temperature difference around heat exchangers is considered 5°C. Therefore *T*_14_ and *T*_18_ is taken accordingly in Eqs. (8) and (9).

(8) $$\begin{array}{c} T_{14}=T_{5}+5{}^{\circ }C\\ T_{14}=63.98{}^{\circ }C+5{}^{\circ }C=68.98{}^{\circ }C \end{array}$$

(9) $$\begin{array}{c} T_{18}=T_{10}-5{}^{\circ }C\\ T_{18}=26{}^{\circ }C-5{}^{\circ }C=21{}^{\circ }C \end{array}$$

Based on the dew point of VOCs exhaust, the temperature of hot air can be reduced up to 85°C.

$$T_{16}=85{}^{\circ }C$$

Mass flow rates of condenser outlet water across heat exchanger H1 and hot air across heat exchanger H2 can be calculated using Eqs. (10) and (11).

(10) $$\begin{array}{c} m_{13}=\;m_{14}=\;\frac{Q_{H1}}{c_{p}\;\left(T_{13}-T_{14}\right)}\\ m_{13}=\;m_{14}=\;\;12\;\text{MW}/\;\left(4180\;J/\text{kg}.^{\circ }C\;\times \;\left(85^{\circ }C-68.98^{\circ }C\right)\right)\;=\;\;179.2\;\text{kg}/s \end{array}$$

(11) $$\begin{array}{ccc} m_{15} & = & \;m_{16}=\;\frac{Q_{H2}}{c_{pa}\;\left(T_{15}-T_{16}\right)}\\ m_{15} & = & \;m_{16}=\;0.38\;\text{MW}\;/\left(1000\;J/\text{kg}.{}^{\circ }C\;\times \;\left(350{}^{\circ }C-85{}^{\circ }C\right)\right)\;=\;1.43\;\text{kg}/s \end{array}$$

The coolant flow rates and heat sink stream flow rates are assumed to be the same as the MD feed flowrate.

$$\begin{array}{cc} m_{10} & =\;m_{11}=185\;kg/s\\ m_{17} & =m_{18}=185\;kg/s \end{array}$$

Thermal power dissipation (*Q_c_*) from the system by cooling water can be calculated using Eq. (12).

(12) $$\begin{array}{cc} Q_{c} & =Q_{H3}=\;m_{10}c_{p}\left(T_{11}-T_{10}\right)\\ Q_{c} & =Q_{H3}=185\;\text{kg}/s\;\times \;4180\;J/\text{kg}.{}^{\circ }C\;C\;\times \;\left(39{}^{\circ }C-26{}^{\circ }C\right)=10\;\text{MW} \end{array}$$

Heat sink stream inlet temperature can be calculated using Eq. (13).

(13) $$\begin{array}{cc} T_{17} & =\;T_{18}\;-\frac{Q_{H3}}{m_{18}c_{p}\;}\\ T_{17} & =21{}^{\circ }C-\;\left(10\text{MW}/\left(185\;\text{kg}/s\;\times 4180\;J/\text{kg}.{}^{\circ }C\right)\right)=\;8{}^{\circ }C \end{array}$$

Thermal power loss (*Q_l_*) can be calculated using Eq. (14)

(14) $$\begin{array}{cc} Q_{l} & =Q_{t}-Q_{c}\\ Q_{l} & =12.38\;\text{MW}-10\;\text{MW}\;=\;2.38\;\text{MW} \end{array}$$

Mass flow rates and temperature of streams results for the integrated system are summarized in Table 1.

**Table 1:** Mass flowrates and temperatures of all main streams involved in the system.

Streams (#)		Mass flowrate, m (kg/s)	Temperature, T (°C)
Makeup wastewater (1)		4.17	20
Wastewater after pre-treatment (2)		4.17	20
Wastewater after mixing tank (4)		185	63.98
Wastewater inlet for heat exchanger H1 (associated with heat source A)	Inlet (5)	179.2	63.98
	Outlet (6)	179.2	80
Wastewater inlet for heat exchanger H2 (associated with heat source B)	Inlet (7)	5.67	63.98
	Outlet (8)	5.67	80
MD feed (9)		185	80
Concentrate/Retentate (3)		180.83	65
Distillate/permeate (12)		4.17	43
Cooling water	Inlet (10)	185	26
	Outlet (11)	185	39
Condenser outlet water	Inlet (13)	179.2	85
	Outlet (14)	179.2	68.98
Hot air	Inlet (15)	1.43	350
	Outlet (16)	1.43	85
Heat sink stream	Inlet (17)	185	8
	Outlet (18)	185	21

## 2.2. Equipment design

The major equipment are MD modules and heat exchangers.

1. Membrane modules and membrane area

Total membrane area (A_T_) can be calculated using Eq. (15).

(15) $$\begin{array}{cc} A_{T} & =m_{dT}\frac{A_{m}}{m_{d}}\\ A_{T} & =4.17\;kg/s\times \left(4.6\;m^{2}/0.0075\;kg/s\right)\;=\;2553\;m^{2} \end{array}$$

Total number of MD cascades = 2557 m^2^/4.6 m^2^ = 555

Total number of MD modules required = 555 × 2 = 1110

2. Heat Exchangers

The required heat exchangers area (A_HX_) can be calculated using Eq. (16).

(16) $$A_{\text{HX}}=\frac{Q_{HX}}{U\Delta T}$$

where subscript X =1,2 and 3, U is overall heat transfer coefficient (for water: 250 W/m^2^.C and for air: 10 W/m^2^.C), and ΔT represents the temperature difference between inlet and outlet streams.

$$\begin{array}{c} A_{H1}=\frac{Q_{H1}}{U_{w}\left(T_{13}-T_{14}\right)}=\text{MW}\;/\;\left(250\;W/m^{2}.C\;\times \;\left(85^{\circ }C\;-68.98^{\circ }C\right)\right)=2997\;m^{2}\\ A_{H2}=\frac{Q_{H2}}{U_{a}\left(T_{15}-T_{16}\right)}=0.38\;\text{MW}\;/\;\left(10\;W/m^{2}.C\;\times \;\left(350^{\circ }C\;-85^{\circ }C\right)\right)=143\;m^{2}\\ A_{H3}=\frac{Q_{H3}}{U_{w}\left(T_{11}-T_{10}\right)}=\;10\;\text{MW}\;/\;\left(250\;W/m^{2}.C\;\times \;\left(39^{\circ }C\;-26^{\circ }C\right)\right)\;=\;3093\;m^{2} \end{array}$$

3. Pumps

Based on the flow rates of streams, commercial pumps/compressors, and water tanks are considersedto operate industrial-scale MD system.
Water pumps: Capacity = 670 m^3^/h, Number of pumps=4
Water pumps: Capacity = 15 m^3^/h, Number of pumps=2
Air compressor: Number of compressors=1

## 2.3. Cost evaluation

The detailed calculation method for capital expenditure (CAPEX) and operating and maintenance expenditure (OPMEX) for a 15 m^3^/h AGMD plant is presented below.

### 2.3.1. Capital investment

Eq. (17) was used for total equipment cost (C_*eq*_) calculation considering the factored estimate method [10].

(17) $$C_{eq}=I{\;}_{CEPCI}\left[\sum {\left(\frac{C_{N}}{C_{R}}\right)}^{m}C_{\text{Pi}}\right]$$

where C_Pi_ represents the reference cost of equipment, C_N_ & C_R_ denote new (desired) and reference capacity of the equipment, I_CEPCI_ is used for the value of the chemical engineering plant cost index (CEPCI). However, m represents the degression constant, and its value is 0.8 for MD modules and heat exchangers, and 0.667 for pumps and water tanks. Table 2 shows the cost of reference equipment.

1. Membrane Distillation modules cost

**Table 2:** Cost of reference equipment.

C_Pi_ of MD modules	6100 $/module [5]
C_Pi_ of MD membranes	90 $/m^2^[11]
C_Pi_ of heat exchangers	325 $/m^2^[12]
C_Pi_ of pumps	5500 $/100 m^3^/h [13]
C_Pi_ of water tanks	130 $/m^3^/day [14]
C_Pi_ of process control	140 $/m^3^/day [14]

Total cost of MD modules = (575/550)^a^ × (1110/1)^0.8^ × $6100 = $1741434

where a is I_CEPCI_ and used to account for inflation. The MD modules were manufactured in 2010, and we used the most recent CEPCI for MD modules to convert all costs to the year 2017.

2. Membranes cost

Total cost of membranes = (2553 m^2^/1 m^2^)^0.8^ × $90 = $47854

3. Heat Exchangers cost

Total cost of heat exchanger H1 = (575/381.1)^b^ × (2997 m^2^/1 m^2^)^0.8^ × $325 = $296414

Total cost of heat exchanger H2 = (575/381.1)^b^ × (143 m^2^/1 m^2^)^0.8^ × $325 = $26046

Total cost of heat exchanger H3 = (575/381.1)^b^ × (3093 m^2^/1 m^2^)^0.8^ × $325 = $303960

Total cost of Heat exchangers = $296414 + $26046 + $303960 = $626420

where b is I_CEPCI_ and used to account for inflation. Cost curves were published in the year 1995, and we used the most recent CEPCI for heat exchangers to convert all costs to the year 2017.

4. Pumps and compressor cost

Total cost of 4 water pumps =(575/389.5)^c^ × (666 m^3^/h/100 m^3^/h)^0.667^ × $5500 × 4 = $115038

Total cost of 2 water pumps = (575/389.5)^c^ × (15 m^3^/h/100 m^3^/h)^0.667^ × $5500 × 2 = $4582

Total cost of 1 air compressor = (575/389.5)^c^ × $23500 =$34692

Total cost of pumps and compressors = $115038+ $4582+ $34692 = $154311

where c is I_CEPCI_ and used to account for inflation. Cost curves were published in the year 1998, and we used the most recent CEPCI for pumps to convert all costs to the year 2017.

5. Water tanks cost

Total MD feed flow rate = Total coolant flow rate = 14652 m^3^/day

Feed and coolant tanks cost = (14652 m^3^/day /1 m^3^/day)^0.667^ × 2 × $130 = $156180

Total permeate flow rate = Total pretreated wastewater flow rate = 330 m^3^/day

Permeate and pre-treatment tanks cost = (330 m^3^/day /1 m^3^/day)^0.667^ × 2 × $130 = $12440

Total tanks cost = $156180+ $12440 = $168620

6. Process control cost

Security controls, sensors and electrical subsystems cost = (14652 m^3^/day /1 m^3^ /day)^0.667^ × $140 = $84097

Purchased equipment cost = $1741434+$47854+$626420+$154311+$168620+$84097= $2822736

*Scenario 1: New wastewater treatment facility*

1. Total depreciable capital (TDC) includes inside and outside battery limits, construction overhead, contingency fees and insurance.

i. Inside battery limits (ISBL) consider total equipment cost and process construction cost, can be calculated by using Eq. (18).

(18) $$\begin{array}{cc} & \text{ISBL}=C_{eq}f_{L}L_{E}\\ & \text{ISBL}=\$2822736\times 5.7^{d}\times 1.2^{e}=\$19307572 \end{array}$$

where d is Lang factor ( f_L_) for the fluid processing plant and e is Location index (L_E_) for Europe.

ii. Outside battery limits (OSBL) consider the storage and administration costs and calculated using Eq. (19).

(19) $$\begin{array}{cc} \text{OSBL} & =0.4\;C_{eq}f_{L}L_{E}\\ \text{OSBL} & =\$19307572\times 0.4=\$7723029 \end{array}$$

iii. Other costs include construction overhead (CO), contingency fees (CF) and insurance (I).

CO = 15% of C_*eq*_ and labor cost
CF = 10% of C_*eq*_
I = 5 % of C_*eq*_
CO = ($2822736+$3600) × 0.15 = $423952
CF = $2822736 × 0.1 = $282274
I = $2822736 × 0.05 = $141137

Total depreciable capital investment (*C_TDC_*) can be calculated using Eq. (20).

(20) $$\begin{array}{c} C_{TDC}=ISBL+OSBL+CO+CF+I\\ C_{TDC}=\$19307572+\$7723029+\$423952+\$282274+\$141137=\$27877964 \end{array}$$

2. Total permanent capital (TPC) includes site preparation and land costs (LC) and plant startup cost (PS).

LC = 2% of *C_TDC_*
PS = 10% of *C_TDC_*
LC = $27877964 × 0.02 = $557559
PS = $27877964 × 0.1 = $2787796

Total permanent capital investment (*C_TPC_*) can be calculated using Eq. (21).

(21) $$\begin{array}{c} C_{TPC}=LC+PS\\ C_{TPC}=\$557559+\$2787796=\$3345356 \end{array}$$

3. Working capital (WC) can be calculated using Eq. (22).

(22) $$\begin{array}{c} C_{WC}=8.3\%\;\text{of}\;\text{OSBL}\\ C_{WC}=\$7723029\times 0.0833=\$643328 \end{array}$$

Thus, total capital investment (TCI) for scenario 1 can be calculated using Eq. (23).

(23) $$\begin{array}{c} C_{TCI1}=C_{TDC}+C_{TPC}+C_{WC}\\ C_{TCI1}=\$27877964+\$3345356+\$643328=\$31866648 \end{array}$$

*Scenario 2: Retrofitted wastewater treatment plant*

Total capital investment (TCI) for scenario 2 can be calculated using Eq. (24)

(24) $$C_{TCI2}=C_{eq}+\;I+C_{R}$$

where *C_R_* is the retrofitting cost which is equal to 4% of *C*_eq_.

Retrofitting cost = $2822736 × 0.04 = $112910

*C*_*TCI*2_ = $2822736+$141136+$112910= $3076791

The annual capital investment (C_a_) can be calculated using Eq. (25)

(25) $$C_{a}=\left(\frac{I{\left(1+i\right)}^{L}}{{\left(1+i\right)}^{L}-1}\right)\;C_{\text{TCI}}$$

where annual interest rate i=5% [15,16]and plant life span = L = 20 years.

$$\begin{array}{c} C_{a1}=\$31866648\times \left(\left(0.05\times {\left(1+0.05\right)}^{20}\right)/\left({\left(1+0.05\right)}^{20}-1\right)\right)=\$2557062\\ C_{a2}=\$3076791\times \left(\left(0.05\times {\left(1+0.05\right)}^{20}\right)/\left({\left(1+0.05\right)}^{20}-1\right)\right)=\$246890 \end{array}$$

Normalized capital investment C_NC_ can be calculated using Eq. (26)

(26) $$\begin{array}{cc} C_{\text{NC}} & =\frac{C_{a}}{m_{dT}}\\ C_{\text{NC}1} & =\$2557062/120000\;m^{3}=21.31\;\$/m^{3}\\ C_{\text{NC}2} & =\$\;246890/120000\;m^{3}=2.06\;\$/m^{3} \end{array}$$

### 2.3.2. Operating and maintenance cost

Annual operating and maintenance cost (OPM_a_) is same for both scenarios and considers the following costs.

1. Thermal energy cost when industrial waste heat is used = 0 $/MWh
2. Specific electrical energy demand for the considered range of MD feed flow rate is mentioned in the reference study (0.35 kWh/m^3^)
3. The other operational costs are taken from the literature as follows.
  i. Service and maintenance = 0.033 $/m^3^ [11] [71]
  ii. Labor = 0.03 $/m^3^ [11,^14^,15]
  iii. Cleaning chemicals =0.0018 $/m^3^ [11,14]
  iv. Pretreatment chemicals (if required) = 0.02 $/m^3^ [17]
  v. Annual membrane replacement =15% of total membrane cost [18]
  vi. Brine disposal = 0.0015 $/m^3^ [19]
  vii. Cooling water =0.02 $/m^3^ of total cooling water [20]

Thermal energy cost = $0
Electricity cost = 0.35 kWh/m^3^ × 0.09 $/kWh × 15 m^3^/h × 8000 h=$3780
Maintenance and service cost = 0.033 $/m^3^ × 15 m^3^/h × 8000 h = $3960
Labor cost = 0.03 $/m^3^ × 15 m^3^/h × 8000 h = $3600
Cleaning chemicals =0.0018 $/m^3^ × 15 m^3^/h × 8000 h = $216
Pretreatment chemical = 0.02$/m^3^ × 15 m^3^/h × 8000 h = $2400
Membrane replacement cost = $47854 × 0.15= $7178
Brine disposal cost = 0.0015 $/m^3^ × 15 m^3^/h × 8000 h = $180
Cooling water cost = 0.02 $/m^3^ × 666 m^3^/h × 8000 h = $106560
OPM_a_= $0+$3780+$7178+$3960+$3600+$180+$216+$2400+$106560 = $127874

Normalized operating and maintenance investment (C_NO_) can be calculated using Eq. (27)

(27) $$\begin{array}{cc} C_{\text{NO}} & =\frac{\text{OP}M_{a}}{m_{dT}}\\ C_{\text{NO}} & =\$127874/120000=1.065\;\$/m^{3} \end{array}$$

### Water treatment cost

Unit water treatment cost can be calculated using Eq. (28)

(28) $$\begin{array}{cc} C_{w} & =C_{\text{NC}}+C_{\text{NO}}\\ C_{w1} & =21.31\;\$/m^{3}+1.06\;\$/m^{3}=22.37\;\;\$/m^{3}\\ C_{w2} & =2.06\;\$/m^{3}+1.06\;\$/m^{3}=3.12\;\$/m^{3} \end{array}$$

## Conclusion

This paper focuses on the process design of a waste heat driven MD system for wastewater treatment in nano-electronics industries. The results show that an MD plant of capacity 15 m^3^/h demands 12.38 MW thermal power, which can be provided with internal waste heat sources in nano-electronics industries. The expected wastewater treatment cost ranges from 3 $/m^3^ (retrofitted facility) to 23 $/m^3^ (new wastewater treatment facility). The cost comparison with competitive technologies (i.e., electrochemical process (59 $/m^3^)) shows the economic superiority of waste heat driven MD system. Moreover, the proposed system has two-folds benefits in environmental aspects: water management and emission control.

## Declaration of Competing Interest

The authors declare that they have no known competing financial interests or personal relationships that could have appeared to influence the work reported in this paper.

## References

[1] Lai C.L., Lin S.H. (2004). Treatment of chemical mechanical polishing wastewater by electrocoagulation: system performances and sludge settling characteristics. Chemosphere.

[2] Gilles D.G., Loehr R.C. (1994). Waste generation and minimization in semiconductor industry. J. Environ. Eng..

[3] Eng C.Y., Yan D., Withanage N., Liang Q., Zhou Y. (2019). Wastewater treatment and recycle from a semiconductor industry: a demo-plant study. Water Pract. Technol..

[4] Alkhudhiri A., Darwish N., Hilal N. (2012). Membrane distillation: a comprehensive review. Desalination.

[5] Woldemariam D., Kullab A., Fortkamp U., Magner J., Royen H., Martin A. (2016). Membrane distillation pilot plant trials with pharmaceutical residues and energy demand analysis. Chem. Eng. J..

[6] Ong C.L., Escher W., Paredes S., Khalil A.S.G., Michel B. (2012). A novel concept of energy reuse from high concentration photovoltaic thermal (HCPVT) system for desalination. Desalination.

[7] Noor I.-, Coenen J., Martin A., Dahl O. (2020). Performance assessment of chemical mechanical planarization wastewater treatment in nano-electronics industries using membrane distillation. Sep. Purif. Technol..

[8] Noor I.-, Coenen J., Martin A., Dahl O., Åslin M. (2019). Experimental investigation and techno-economic analysis of tetramethylammonium hydroxide removal from wastewater in nano-electronics manufacturing via membrane distillation. J. Memb. Sci..

[9] Noor I.-, Martin A., Dahl O. (2020). Techno-economic system analysis of membrane distillation process for treatment of chemical mechanical planarization wastewater in nano-electronics industries. Sep. Purif. Technol..

[10] Green D.W., Perry R.H. (2007). Perry's Chemical Engineers’ Handbook. https://books.google.se/books?id=tH7IVcA-MX0C.

[11] Drioli E., Curcio E., Di Profio G., Macedonio F., Criscuoli A. (2006). Integrating membrane contactors technology and pressure-driven membrane operations for seawater desalination: energy, exergy and costs analysis. Chem. Eng. Res. Des..

[12] Hewitt G.F., Pugh S.J. (2007). Approximate design and costing methods for heat exchangers. Heat Transf. Eng..

[13] H.P. Loh, J. Lyons, C.W. White, Process Equipment Cost Estimation, 2002. Report No. DOE/NETL-2002/1169.

[14] K.K. Sirkar, L. Song, Pilot-scale studies for direct contact membrane distillation-based desalination process, 2009.

[15] Kesieme U.K., Milne N., Aral H., Cheng C.Y., Duke M. (2013). Economic analysis of desalination technologies in the context of carbon pricing, and opportunities for membrane distillation. Desalination.

[16] Wade N.M. (2001). Distillation of plant development and cost update. Desalination.

[17] ICIS (2019). Indicative Chemical Prices, Chem. Ind. News. https://www.icis.com/explore/commodities/chemicals/channel-info-chemicals-a-z/.

[18] Medesol Report, Performance and cost estimations of final industrial-size of MEDESOL-2 technology, 2010.

[19] Schwantes R., Chavan K., Winter D., Felsmann C., Pfafferott J. (2018). Techno-economic comparison of membrane distillation and MVC in a zero liquid discharge application. Desalination.

[20] Turton R., Bailie R.C., Whiting W.B., Shaeiwitz J.A. (2009). Analysis, Synthesis, and Design of Chemical Processes. https://books.google.se/books?id=YEJHAQAAIAAJ.

